# Long-term all-optical interrogation of cortical neurons in awake-behaving nonhuman primates

**DOI:** 10.1371/journal.pbio.2005839

**Published:** 2018-08-08

**Authors:** Niansheng Ju, Rundong Jiang, Stephen L. Macknik, Susana Martinez-Conde, Shiming Tang

**Affiliations:** 1 Peking University School of Life Sciences, Beijing, China; 2 Peking-Tsinghua Center for Life Sciences, Beijing, China; 3 IDG/McGovern Institute for Brain Research at Peking University, Beijing, China; 4 Key Laboratory of Machine Perception (Ministry of Education), Peking University, Beijing, China; 5 State University of New York, Downstate Medical Center, Brooklyn, New York, United States of America; Albert Einstein College of Medicine, United States of America

## Abstract

Whereas optogenetic techniques have proven successful in their ability to manipulate neuronal populations—with high spatial and temporal fidelity—in species ranging from insects to rodents, significant obstacles remain in their application to nonhuman primates (NHPs). Robust optogenetics-activated behavior and long-term monitoring of target neurons have been challenging in NHPs. Here, we present a method for all-optical interrogation (AOI), integrating optical stimulation and simultaneous two-photon (2P) imaging of neuronal populations in the primary visual cortex (V1) of awake rhesus macaques. A red-shifted channel-rhodopsin transgene (ChR1/VChR1 [C1V1]) and genetically encoded calcium indicators (genetically encoded calmodulin protein [GCaMP]5 or GCaMP6s) were delivered by adeno-associated viruses (AAVs) and subsequently expressed in V1 neuronal populations for months. We achieved optogenetic stimulation using both single-photon (1P) activation of neuronal populations and 2P activation of single cells, while simultaneously recording 2P calcium imaging in awake NHPs. Optogenetic manipulations of V1 neuronal populations produced reliable artificial visual percepts. Together, our advances show the feasibility of precise and stable AOI of cortical neurons in awake NHPs, which may lead to broad applications in high-level cognition and preclinical testing studies.

## Author summary

This report details the first successful application of long-term all-optical interrogation techniques in monkeys. We have overcome obstacles that prevented the combination of single- and two-photon (1P and 2P) optogenetic stimulation with 2P imaging in awake-behaving monkeys, retesting targeted individual cells and neuronal ensembles over periods that extended beyond 6 months. Our strategy results in repeatable primary visual cortex (V1) neuronal stimulation of the same neurons and produces reliable visual percepts, which monkeys report behaviorally in a visual–motor task. The animals’ behavioral responses to their optogenetic-induced perceptions are comparable to their responses to real visual stimulation. These technical advances establish the feasibility of combined long-term optogenetic manipulation and 2P imaging of neocortical neurons in awake-behaving monkeys. Our approach may be applied to investigate the molecular and circuit-level mechanistic pathways that are unique to primate neural function. These methods also provide a roadmap for preclinical testing of human optogenetic therapies and may serve as the basis for optogenetic studies involving sensorimotor functions relevant to human perception, cognition, behavior, and neurological/psychiatric disorders.

## Introduction

Optogenetic techniques enable the functional characterization of neuronal populations and circuits with high spatial and temporal precision [1–7]. Though relatively understudied as compared to rodents, optogenetics techniques have been applied to the study of high-level cognition circuits in NHPs [8–13], including those underlying human neurological and psychiatric disorders [14–16], and they hold the potential to unveil the mechanistic pathways for visual processing circuits that are found only in humans and NHPs (as the only mammals with retinal foveas) [17,18]. NHP studies are moreover essential for preclinical testing of optogenetic therapies before they can be translated to human applications [11,19,20].

Previous research has recorded optogenetic activation using traditional electrophysiological techniques. This approach is limited, however, because repeated electrode recordings in the same neurons are difficult to achieve across recording sessions in NHPs. In addition, examining opsin expression patterns in vivo within the area targeted by viral vector infusions, while maintaining the health of the neurons, is not currently possible without 2P laser-scanning microscopy [14,21–24]. These combined hurdles call for an all-optical interrogation (AOI) approach to the application of optogenetic methods in NHPs.

AOI is achieved by the combination of optogenetics to perturb neuronal activity, while using calcium or voltage indicators—rather than electrode-based stimulation and recording—to minimize the invasiveness of the readout [25–30]. AOI’s implementation thus allows the monitoring of large neuronal populations—repeatedly and less invasively—with single-cell resolution [31–33], while enabling detailed mapping of neural circuits during behavior [34,35]. Pioneering efforts to apply AOI in NHPs combined optogenetics with both in vivo epifluorescence imaging and intrinsic signal optical imaging [19]. Whereas these techniques allowed for large-field viewing, the spatial resolution of the readout was limited, and specific neurons of interest could not be interrogated repeatedly across recording sessions.

Here, we combined wide-field single-photon (1P) and single-cell 2P optogenetic stimulation techniques with recently developed 2P imaging technique in awake macaques [4,36] to achieve AOI in NHPs. A red-shifted opsin ChR1/VChR1 (C1V1) and calcium indicators GCaMP5G/GCaMP6s were delivered into V1 with adeno-associated viruses (AAVs) and expressed in V1 neuronal populations. The labeled V1 neurons exhibited consistently robust responses, over several months, to either optogenetic or visual stimulation. The behavioral experiments confirmed that robust artificial visual perception could be induced by optogenetic stimulation of V1 neuronal populations.

## Results

### Transgene delivery and expression

We infected area V1 neurons in three monkeys with C1V1 (AAV9–CamKIIα–C1V1(T/T)–ts–EYFP)—a red-shifted channel-rhodopsin transgene—and GCaMP5G/GCaMP6s (AAV1–hSyn-GCamP5G/AAV1–Syn–GCamP6s)—calcium indicators of activity. Six weeks after virus injection, a 1-cm–diameter round optical window (glass coverslip attached to a titanium ring) was implanted onto the cortical surface using dental acrylic cement attached to the bone surrounding the craniotomy. To enhance the stability of 2P imaging, we used a three-point head-fixation design, with two head posts implanted on the forehead of the skull and one on the back. A T-shaped steel frame was connected to these head posts for head stabilization during subsequent imaging and stimulating sessions [36].

**Fig 1:**
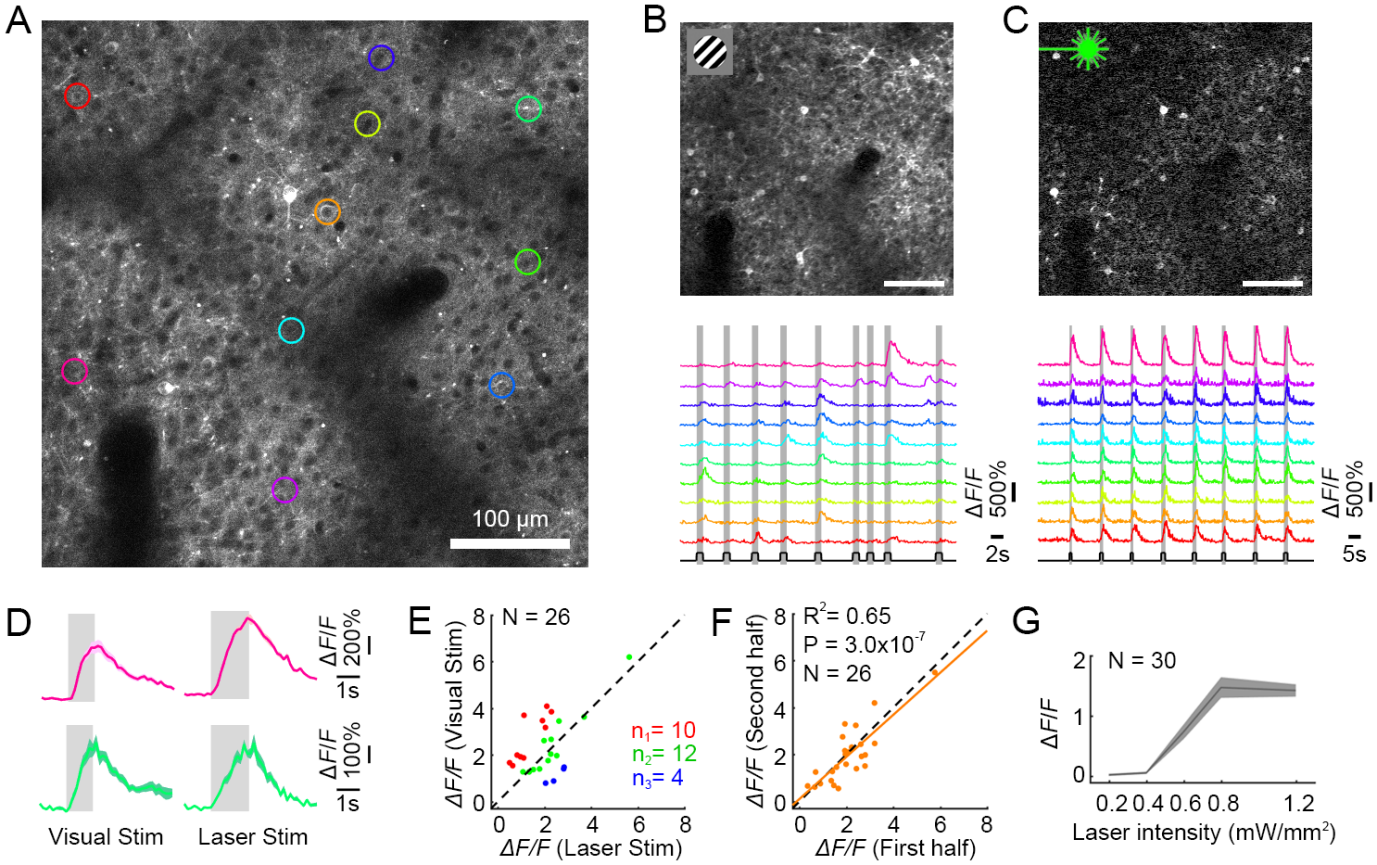
AOI of a V1 neuronal population in awake macaque. (A) 2P image of V1 neurons expressing C1V1–ts–EYFP and GCaMP6s. The colored regions of interest (ROIs) indicate neurons that responded to both visual and optical stimuli, targeted for further analysis. (B) Top, a differential image of GCaMP6s fluorescence (stimulated-baseline [F-F0], averaged across all stimulations), driven by visual stimuli consisting of gratings or colored patches. Bottom, calcium signals from 10 neurons (colors from panel A) in response to 9 varied visual stimuli (presentation times in gray). (C) Top, widefield optogenetic stimulation (0.8 mW/mm^2^, 30 Hz and 25% duty ratio) evoked robust responses in the same neurons. Bottom, 8 sequential identical optogenetic stimulations evoked equivalent responses in each cycle. (D) Responses of two neurons to their preferred visual stimuli (Left pink cell, color patch response; Left green cell, drifting grating response; mean ± s.e.m., *n* = 10 trials) versus photostimulation in the same cells (Right, mean ± s.e.m., *n* = 9 trials). (E) Neuronal population responses (*N* = 26) to their preferred visual stimuli versus laser stimulation. Green dots stand for cells that have similar responses to visual versus laser stimuli (*p* > 0.05), red dots for cells that have significantly stronger responses to visual stimuli versus laser stimulation (*p* < 0.05), and blue dots vice versa (*p* < 0.05). (F) Neuronal population responses (*N* = 26) from the first versus last four trials from a single experiment. (G) Laser intensity dose-response curve (mean ± s.e.m., *n* = 9 trials, *N* = 30 neurons). Optogenetic stimulation saturates at approximately 0.8 mW/mm^2^. Data can be found at https://github.com/EastRainju/Opto-TP. See also S2 Fig. 1P, single-photon; 2P, two-photon; AOI, all-optical interrogation; C1V1, ChR1/VChR1; GCaMP, genetically encoded calmodulin protein; ROI, region of interest.

We imaged layer II/III neurons in the infected cortical area using 2P (Fig 1A). Dark cell bodies indicate that C1V1–ts–EYFP expression was localized to the membrane [37] (see also S1 Fig). Fluorescence of GCaMP6s was relatively weak in the absence of cellular responses to either visual or optogenetic stimulation.

### Simultaneous optical manipulation and 2P readout

NHPs maintained fixation while visual stimuli consisting of drifting gratings and color patches were presented sequentially on the neuronal receptive field for 1 second, with >2-second interstimuli intervals. We recorded robust neuronal calcium responses that showed normal orientation and color selectivity, as well as well-organized receptive-field spatial organization (Fig 1B, S3 and S4 Figs).

We then stimulated the neurons optogenetically. Using 1P stimulation (532-nm laser), we illuminated the entire imaging field (a 1-mm^2^ laser spot) while measuring neuronal activity simultaneously with 2P imaging. Simultaneous stimulation/imaging presented a significant challenge, because—although the stimulation and recording wavelengths were sufficiently separated and filtered optically—the optogenetic stimulation power was orders of magnitude higher than the fluorescence power emitted by the activated cells. Thus, stimulation light leaked through the filters and into the highly amplified photomultipliers (PMTs), with higher power than the relatively small GCaMP fluorescence signal. Therefore, the full-field optogenetic stimulation laser was powered down whenever each 2P imaging scan targeted the central 75% of the FOV (24 ms out of each 32-ms imaging scan frame). Thus, the entire field was stimulated for 8 ms out of every 32-ms scan (25% duty-cycle stimulation at 31.25 Hz). This allowed us to view the optogenetic activation responses artifact-free (S6 Fig).

**Fig 2:**
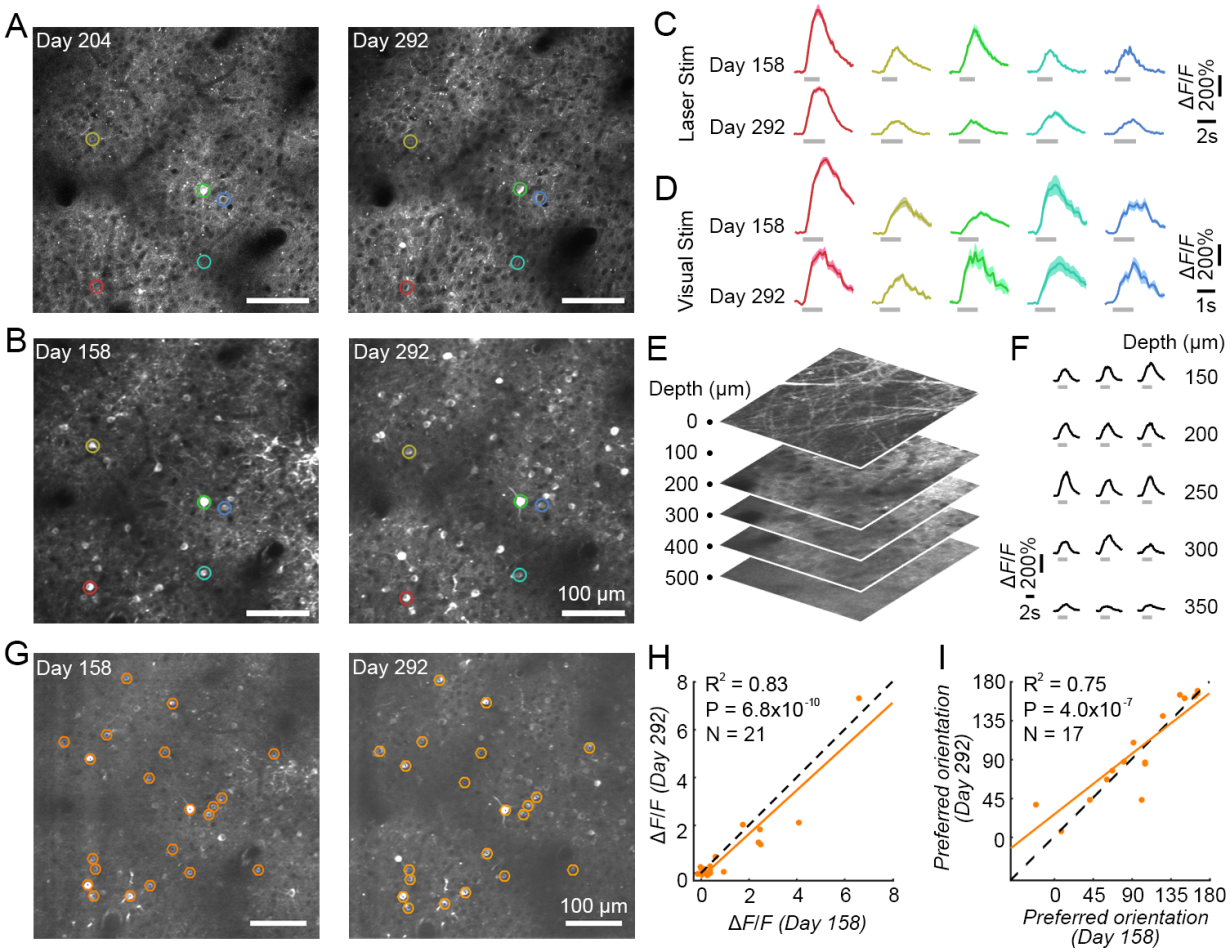
Long-term stability of AOI in macaque V1. (A) 2P images of a V1 neuronal population on Day 204 and Day 292 after virus injection. (B) The same neuronal population was activated with visual stimuli on Day 158 and Day 292. (C) Repeated optical stimulation evoked stable neuronal activity in 5 example cells (colored circles in A-B) on Day 158 and Day 292 (mean ± s.e.m., *n* = 9 trials). Laser stimulation was identical to Fig 1. (D) Visual stimulation also evoked repeatable neuronal activity in the same 5 cells (from A-C) on Day 158 and Day 292 (mean ± s.e.m., *n* = 10 and 5 trials, respectively). (E) Multilayer 2P volume from the cortical surface to 500-μm depth. (F) Optogenetic calcium responses in different layers (five layers from 150-μm to 350-μm depth, three neurons from each layer) under 1P stimulation. (G) 2P images averaged across photostimulation experiments on Days 158 and 292, respectively. The orange ROIs indicate neurons that were activated by both visual and laser stimulation, on both recording days, targeted for further analysis. (H) Neuronal response correlations to optical stimulation on Days 158 and 292. Each point represents a single neuron, and the orange solid line is their corresponding regression line. The dashed line is the unity line. R^2^ = 0.83 when fitted to the unity line, indicating that there was no significant difference between responses with long-term repeated stimulation. (I) Neuronal orientation tuning correlations on Days 158 and 292. The orientation tuned neurons were picked by ANOVA with *P* < 0.05. Data can be found at https://github.com/EastRainju/Opto-TP. 1P, single-photon; 2P, two-photon; AOI, all-optical interrogation; ROI, region of interest; V1, primary visual cortex.

**Fig 3:**
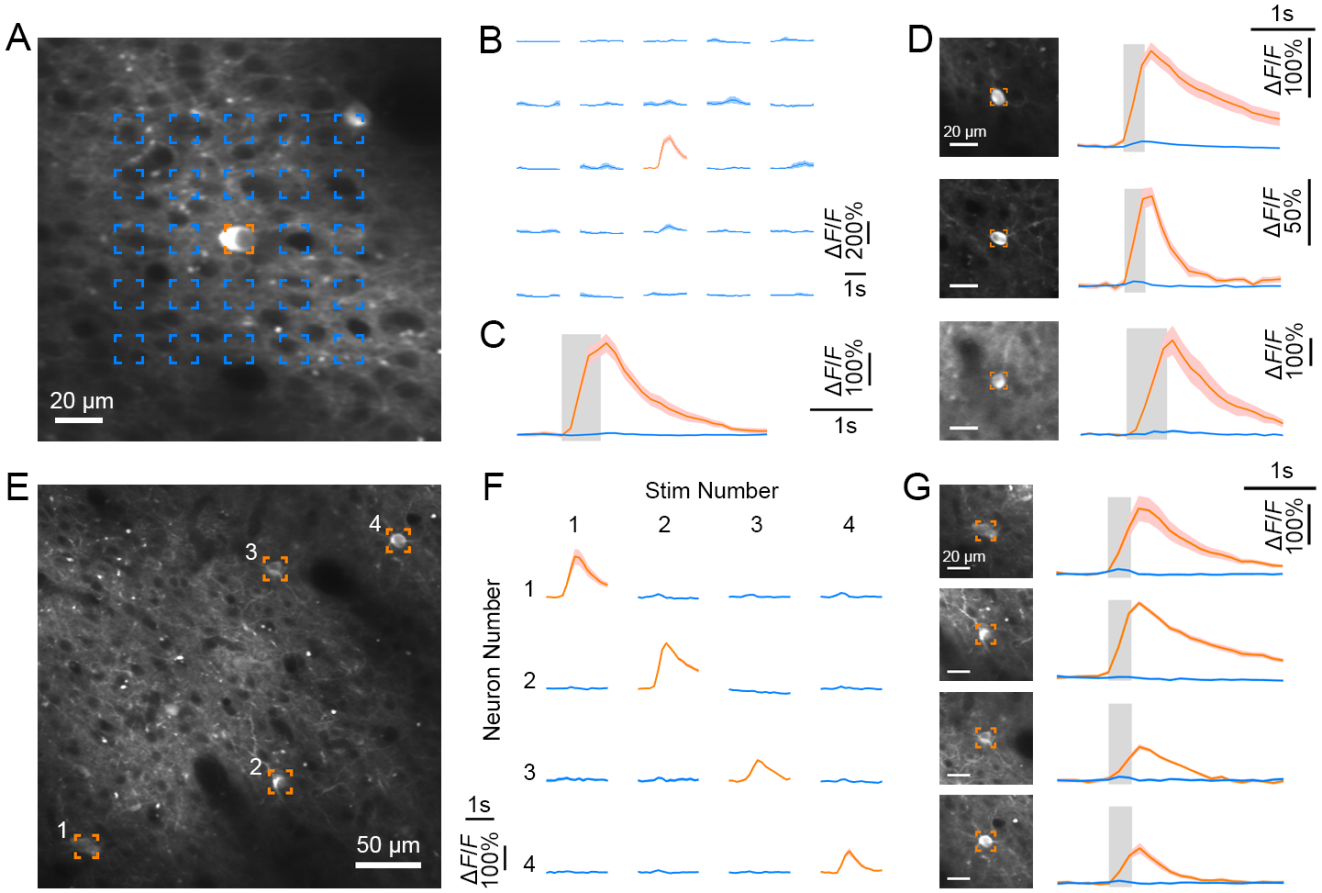
AOI with single-cell resolution in V1. (A) 2P image of a targeted neuron co-expressing GCaMP5G and C1V1 (orange box, on Day 400 at 161 μm). (B) Calcium responses of the targeted neuron following spiral 2P optogenetic stimulation at each of the 5 × 5 grid locations from (A). The target neuron responded only to directly focused 2P stimulation, indicating that 2P stimulation is spatially precise. (C) Average fluorescence traces from the neuron when targeting 2P stimulation directly at the soma (orange) versus the surrounding parenchyma (blue; mean ± s.e.m., *n* = 15 trials). (D) Fluorescence traces of three other neurons under 2P stimulation (first two on Day 570 and third on Day 400). (E) 2P image containing four targeted neurons co-expressing GCaMP5G and C1V1 (on Day 570 at 189 μm). (F) Calcium responses of the targeted neurons (from E) when they were sequentially activated with spiral 2P stimulation. Each row demonstrates one of four neurons’ activity (numbered as in E) during stimulation on either the same or alternate numbered neuron (columns). (G) 2P images of the targeted neurons (E) and their individual average fluorescence traces when each of them was targeted (orange) versus not targeted (blue; mean ± s.e.m., *n* = 10 trials). Data can be found at https://github.com/EastRainju/Opto-TP. 2P, two-photon; AOI, all-optical interrogation; C1V1, ChR1/VChR1; GCaMP, genetically encoded calmodulin protein; V1, primary visual cortex.

The cells that were successfully stimulated optogenetically constituted a considerable fraction of the targeted population and responded vigorously (Fig 1C). By repeatedly stimulating—both optogenetically and visually—we made three observations: 1) responses from the two modes of stimulation were comparable to each other in both amplitude and dynamics (Fig 1D and 1E); 2) repeated stimulation resulted in similarly sized responses (Fig 1F); 3) optogenetic activation did not alter the receptive field properties of neurons that were subsequently stimulated with visual stimuli (S3A Fig). Notably, the dose-response curve revealed that the average laser-evoked responses were saturated at approximately 0.8 mW/mm^2^, indicating high sensitivity of the optical manipulation system (Fig 1G and S2 Fig).

### Assessment of long-term stability

Using AOI, we assessed the long-term stability of both transgene expression and the physiological response strength to visual and optogenetic stimulation in the behaving NHPs. Transgene expression level and pattern were maintained (Fig 2A), and neurons exhibited consistently robust responses and tuning to visual stimuli (Fig 2B, 2D and 2I, S3 Fig from monkey M1 and S4 Fig from monkey M3) over several months. The same neuronal population was also repeatedly and stably activated by optogenetic stimulation over a 4-month period (Fig 2C, 2G and 2H). We also evaluated the transgene expression at different cortical depths, from the surface to 500 μm. At 10 months post-infection, there was abundant expression between 150 to 300 μm (Fig 2E and S5 Fig), and neurons in this depth range responded robustly to optogenetic stimulation (Fig 2F). Thus, both expression level and optogenetic responses remained stable over long time periods (in our experience, 6 months or more) in NHP cortex.

### All-optical single-cell resolution stimulation and recording

A powerful way to assess neural circuit function is to photostimulate an individual neuron (minimizing stimulation of unwanted targets) while simultaneously monitoring the activity of the connected neurons in the network [5,16,35]. To perform simultaneous single-cell–resolution 2P optogenetic activation with 2P calcium imaging of the neuronal population, we added a second optical path to our microscope—driven by a mode-locked femtosecond laser (λ = 1070 nm, 50 fs)—and applied 2P stimulation with spiral galvanometer scanning targeted to the somas of the target cells [34].

To examine the spatial specificity of 2P activation, we measured the calcium response of the targeted neuron as a function of multiple stimulation sites (5 × 5 grid) (Fig 3A) [34,35]. We sequentially stimulated each of the sites using 2P spiral activation. Robust responses in the central neuron were evoked only when the target neuron was directly targeted (Fig 3B–3D), suggesting that spiral 2P stimulation has high spatial precision and must be focused on the neuron for strong optogenetic activation to occur. We then simultaneously monitored and sequentially manipulated several neurons in one imaging field (Fig 3E). Each of these neurons generated strong responses only when targeted by the 2P activation laser (Fig 3F and 3G).

### Optogenetic manipulation of behavior

**Fig 4:**
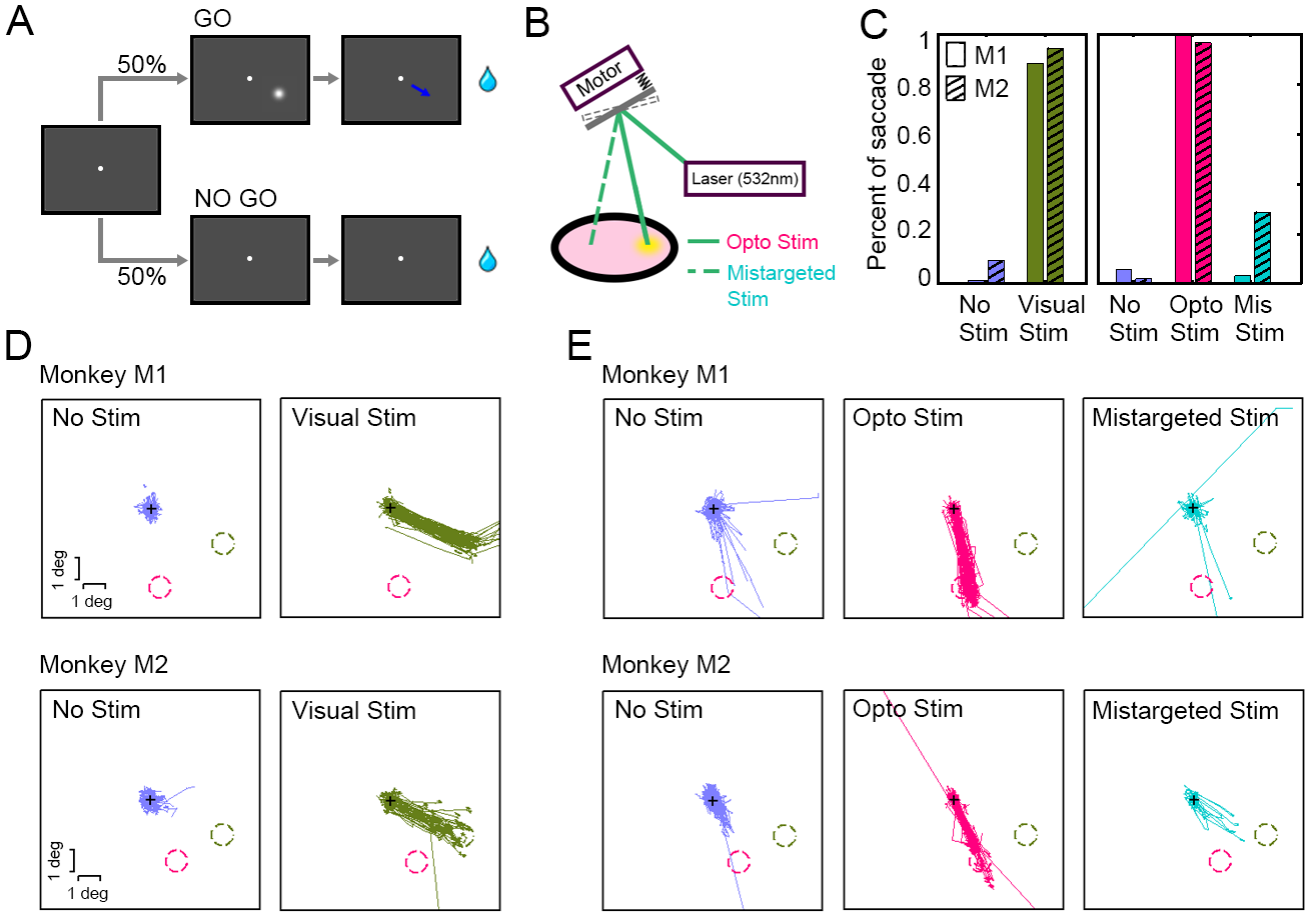
Behavior induced by visual versus optogenetic stimulation in V1. (A) “GO”/“NO GO” visual object detecting task. Monkeys were trained to report the onset of a visual cue (either a visual stimulus—Visual Stim—or an artificial visual perception induced by optogenetic stimulus—Opto Stim) by producing an eye movement. Each trial began when the NHP fixated the central fixation point. In the GO condition, monkeys were required to make a saccade within 500 ms of the cue onset to obtain a juice reward. In the NO GO condition, no stimulus was presented, and monkeys were tasked with maintaining fixation for 2,000 ms to get a juice reward. (B) An apparatus used for redirecting laser pulses towards a nearby cortical area not transduced with C1V1 (Mistargeted Stim, see insert). (C) Analysis of behavior in two monkeys (M1 & M2) during the Visual Stim block (No Stim [blue] and Visual Stim [green]) versus the Opto Stim block (No Stim [blue], Opto Stim [red], and Mistargeted Stim [teal]). The trial numbers that we recorded for the five conditions (No Stim, Visual Stim, No Stim, Opto Stim, Mis Stim) for Monkey M1 were {70, 94, 244, 96, 62}, respectively, and {32, 50, 139, 51, 14} for Monkey M2, respectively. (D) Saccadic trajectories in the Visual Stim block. The green dashed circles indicate the location of the Visual Stim, and the red dashed circles denote the receptive fields of C1V1-expressing sites. (E) Saccadic trajectories in the Opto Stim block. With Opto Stim, both monkeys uniformly targeted their eye movements to the receptive fields of the C1V1-expressing site. Data can be found at https://github.com/EastRainju/Opto-TP. C1V1, ChR1/VChR1; NHP, nonhuman primate; PMT, photomultiplier; V1, primary visual cortex.

To assess the monkeys’ perception from optogenetic stimulation of V1 neuronal populations, we designed a “GO”/“NO GO” visual object detection task, in which two monkeys were required to report the appearance of a visual cue using eye movements (Fig 4A).

Each trial began when the NHP fixated the central fixation point. Subsequently, a 0.5-degree Gaussian white dot was presented for 22 ms at an eccentricity of approximately 3 degrees as a visual cue for GO (an eye fixation break), and the NHP was rewarded for producing a saccade within 500 ms. On the NO GO trials (50%, no visual cue), the animal was rewarded for holding fixation for 2,000 ms for the entire trial. Training proceeded until the NHPs conducted this task with high accuracy (>80% correct rate; Visual Stim; Fig 4C). Notably, both monkeys tended to make eye movements towards the location of the visual cues (Fig 4D, green; SD of saccade endpoints from the target: 0.22 and 0.69 degrees for Monkey M1 and M2, respectively), though any saccade exceeding 1 degree in magnitude was sufficient to receive a reward.

We then examined the artificial visual perception generated by optogenetic stimulation—Opto Stim. The GO condition here had no visual cue. Instead, we conducted optogenetic stimulation (a 532-nm, 66-ms laser pulse, subtending 1 mm^2^ for Monkey M1, and a 15-Hz, 33% duty-cycle [22 ms on, 44 ms off], 0.8-mW laser pulse train for Monkey M2) at the position of the C1V1-expressing cortex (about 3 degrees eccentric from the fovea, in a different position from the stimulus in the Visual Stim block, so that saccadic targeting would indicate the monkey’s differential perceived stimulation within visual space). Similar to the Visual Stim condition, monkeys in Opto Stim received a juice reward if they produced a saccade (>2 degrees) after the optogenetic stimulation. Both monkeys performed this task well after 3–5 sessions as a result of Opto Stim, with 99% versus 96% accuracy for Monkeys M1 versus M2, respectively (Opto Stim; Fig 4C). The eye movements correctly targeted the stimulation locations within visual space, corresponding to the retinotopic C1V1-expressing loci (which were never otherwise targeted with Visual Stim cues; SD of saccade endpoints from the target: 0.33 and 0.51 degrees for Monkey M1 and M2, respectively). This further confirmed that optogenetic stimulation successfully induced artificial visual perception in the NHPs (Opto Stim; Fig 4E).

To rule out the possibility that any of the observed effects were due to artifacts resulting from the physical side effects of Opto Stim, we interleaved Mistargeted Stim trials (8.3%) in the GO condition: Here, we redirected the laser to a region of V1 cortex that did not express C1V1 (Fig 4B). This mistargeted laser should not have been capable of evoking either optogenetic activation of neurons or artificial visual perception. This control condition was treated as a GO task, and monkeys were again rewarded for saccades in any direction, launched immediately after laser onset (<500 ms). Despite this incentive, we observed significantly fewer saccades in the control condition (*p* < 10^−20^ for Monkey M1 and *p* < 10^−10^ for Monkey M2) (Opto Stim versus Mistargeted Stim; Fig 4C), indicating that the monkeys were truly not aware of the mistargeted laser stimulation. Note that we sometimes observed saccades in the “No Stim” period before the “Opto Stim” block that were biased slightly toward the optogenetic target area, perhaps because perception induced by our Opto Stim condition was weaker than from Visual Stim and thus the monkeys were more likely to guess. But in general, the percentage saccades launched was much lower in the “No Stim” condition.

**Fig 5:**
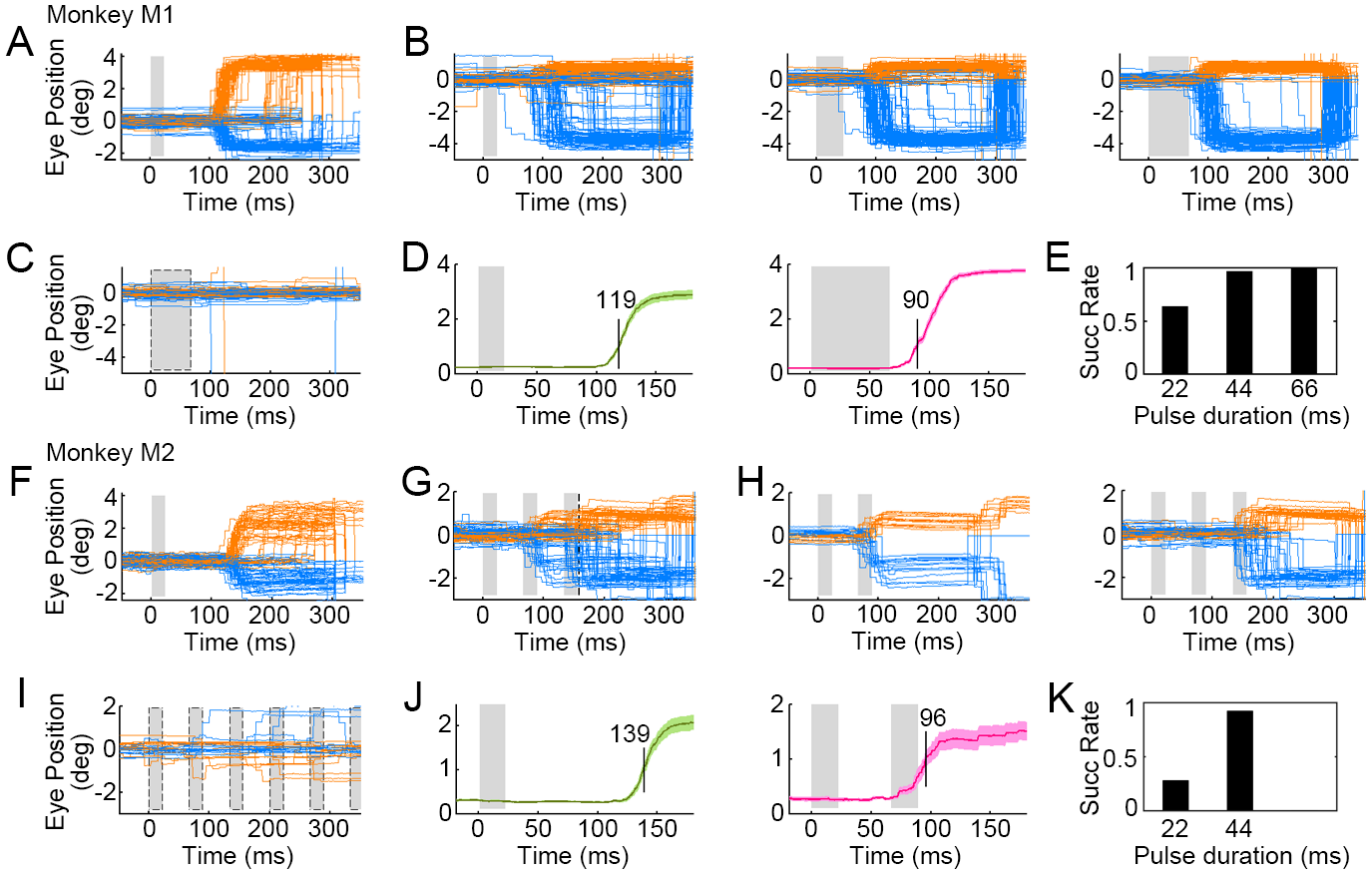
Latencies of saccades triggered by optogenetic activation versus visual stimulation. (A) Saccades triggered by visual stimuli (single 22-ms flash) in Monkey M1. The gray area denotes the stimulation period. (B) Saccades triggered by Opto Stim (single laser pulse with durations of 22, 44, and 66 ms, respectively) on the target (C1V1-expressing) cortical area of Monkey M1. Orange and blue lines represent horizontal and vertical eye positions, respectively. (C) Eye traces when the stimulation laser (66-ms pulse) was targeted to an unlabeled region of cortex (Mistargeted Stim). (D) Average saccade distance to the fixation point in response to visual (Left, green curve) versus optogenetic (Right, red curve) stimulation (gray denotes stimulation period; mean ± s.e.m., *n* = 94 and 96 trials, respectively). Visually induced saccades crossed the 1-degree threshold with an average latency of 119 ms, whereas Opto Stim–induced saccades crossed the 1-degree threshold with an average latency of approximately 90 ms. (E) Success rates of reactions under Opto Stim with varying laser pulse durations. (F) Saccades triggered by visual stimuli (single 22-ms flash) in Monkey M2. (G) Saccades triggered by Opto Stim (laser pulse train, at 15 Hz, 22 ms, 0.8 mW) on the target (C1V1-expressing) cortical region. (H) Saccades during Opto Stim can be sorted into two groups as a function of latency. (Left) Saccades driven by the first laser pulse of 22 ms (latency: approximately 96 ms). (Right) Saccades that failed to initiate on first pulse but instead initiated to the second laser pulse. (I) Eye traces when the stimulation laser pulses were targeted to an unlabeled region of cortex. (J) Average saccade distance to the fixation point in response to Visual Stim (Left, green curve; mean ± s.e.m., *n* = 50) versus Opto Stim (Right, red curve; mean ± s.e.m., *n* = 15). (K) Saccadic stimulation success rates as a function of Opto Stim versus cumulative pulse durations. Data can be found at https://github.com/EastRainju/Opto-TP. C1V1, ChR1/VChR1.

We also studied saccadic latencies as a function of stimulus type and duration. For Visual Stim, saccadic responses were swift and robust (Fig 5A), and exhibited consistent latencies of approximately 119 ms, measured as the time between cue onset and the saccade crossing the 1-degree magnitude threshold (Fig 5D). During Opto Stim (2.4 mW/mm^2^), we found that laser pulses of 44-ms duration (or more) elicited robust responses (Fig 5B). For Monkey M2, similar results were found (Fig 5F–5K). Two pulse stimulation (44-ms total duration) induced responses in 92% trials (Fig 5K) in Monkey M2, similar to Monkey M1 under 44-ms duration single-pulse photostimulation. Saccadic latencies from optogenetic stimulation in both monkeys was 30–40 ms shorter than from visual stimulation, averaging about 90 ms after laser onset (Fig 5D and 5J). This 30–40 ms difference arose presumably because optogenetic stimulation bypassed the subcortical visual pathway. This observation is consistent with previous studies of visual signal propagation from the retina to V1 [38,39].

## Discussion

Optogenetic applications in NHPs have facilitated our understanding of sensory processing, decision making, and the bases of cognition [9,10,14,21,40] and will likely play a key role in future brain–computer interfaces, neural prosthetics, and methods to counteract cognitive decline in the aging human brain. As such, optogenetic techniques are undergoing rapid translation to human clinical use. A critical step in the approval, implementation, and efficacy of optogenetic therapies will be preclinical testing in NHPs, for which methods are currently lacking.

Here, we combined optogenetic stimulation with 2P calcium imaging of neuronal responses to achieve AOI in awake-behaving macaque monkeys, in which we co-infected V1 neurons with C1V1 and GCaMP6s and monitored calcium signals using 2P microscopy while stimulating optogenetically. Our experiments revealed consistently robust neuronal responses to both visual and optogenetic stimulation over many months (Figs 1 and 2). 2P optogenetic stimulation also evoked strong neuronal responses with targeted single-cell resolution (Fig 3). Optogenetic milliwatt-level stimulation in V1 cells produced strong and specific responses in functionally identified visual cells. Finally, we compared optogenetically derived to visually derived perception by assessing the dynamics of saccadic eye movements produced in response to both modes of stimulation (Figs 4 and 5). Together, the above results demonstrate the high sensitivity and stability of our AOI strategy.

### Expression of optogenetic actuator and calcium indicator in monkey cortex

Channelrhodopsin-2 (ChR2) is a commonly used optogenetic actuator for NHPs, though it often requires high laser power to evoke neuronal and behavioral responses [12–15,21]. The high conductance and red-shifted absorption spectrum of C1V1 makes it a preferable choice [10,37,41,42]. This is especially true for AOI experiments, since C1V1’s excitation spectrum is well separated from that of GCaMPs [34,35]. Expression of C1V1–ts–EYFP was robust, and we observed membrane-localized EYFP fluorescence, which has previously indicated membrane localization of C1V1 [41] (Fig 1 and S1 Fig). We visualized GCaMP6s fluorescence and filtered the widefield 1P stimulation pulses with a 500 ± 12.5-nm filter to block most of the EYFP fluorescence. Although the imaging quality was somewhat reduced due to the filter, we nevertheless identified robust responses derived from both visual and optogenetic stimulation (Figs 1 and 2). Note that we did not achieve high efficiency of co-expression of C1V1–ts–EYFP and GCaMP6s in single neurons, and we found that many neurons could be activated by our wide-field illumination but not by our single-cell photostimulation.

Precise quantification of the single-cell expression levels was not possible with our methods because the bright background fluorescence in our approach was likely contributed to by fluorescence of other neurons due to the membrane-bound targeting of the specific indicator we chose. Dendrites from other neurons—and even the soma membranes of directly abutting neurons—could not be perfectly isolated from any given target neuron. We expect that this issue of precise single-cell quantification would be ameliorated by using an indicator that expresses within the cytosol (labeling the cell body only) rather that the membrane. This is why we also tested C1V1–porcine teschovirus-1 2A (P2A)–mCherry, with the hope that the mCherry would express inside the cytosol of cell bodies, thus allowing direct quantification of single-cell fluorescence. Alas, the efficiency of photoactivation of this construct was much lower than the C1V1–ts–EYFP, for unknown reasons. We are thus currently working to develop soma-targeted C1V1-EYFP for both high efficiency photoactivation and quantification of expression [5]. We conclude that more powerful molecular tools and gene delivery techniques further advance their utility in NHPs.

### Minimally invasive AOI of neuronal populations in behaving monkeys

AOI using C1V1 results in much lower tissue damage than what would be caused by repeated probe penetration, or from the photodamage expected with ChR2 constructs [16,20]. A primary limitation of our method arose from the 2P imaging-depth limit. This confined our AOI to superficial cortical circuits, lying within 500 μm of the surface [43]. New multiphoton microscopy methods will improve and extend the depth limit of AOI to as deep as 1 mm [44]. Cellular-resolution imaging of subcortical structures is currently achievable with fiber-optic confocal laser endomicroscopy (CLE) techniques [45,46].

Because the co-expression level of C1V1 and GCaMP was low in our experiments, it is unlikely that we accidentally stimulated unseen dendrites of untargeted neurons while stimulating our target neurons. But this problem—unwittingly stimulating unwanted hidden dendrites that drive neurons other than the targeted neurons—will rise in significance as expression density improves. Soma-targeted opsins serve to minimize this concern [5], which is why we are currently working to develop soma-targeted C1V1–EYFPs that could improve specificity of 2P stimulation.

### Behavior effects induced by optical stimulation

Electrical microstimulation of the visual cortex evokes phosphene perception in humans, as well as saccadic eye movements in NHPs [39,47–50]. Similarly, optical stimulation of monkey V1 has been reported to induce saccades [13], which we also observed. One refinement of our current design over prior work was to include a control condition in which we targeted an unlabeled region of cortex to rule out potential non-optogenetic artifacts related to laser activation. Interestingly, the animals did not immediately respond to Opto Stim when switching from the Visual Stim block. Though both monkeys required fewer than 30 trials to first detect the Opto Stim, this could indicate that the percept derived by the Opto Stim was not identical to that derived from the Visual Stim. If so, the monkeys might have generalized their initial responses to novel stimuli (triggered by the Opto Stim), in much the same way as they might do during operant conditioning of an unfamiliar visual stimulus. Moreover, we discovered that saccadic responses were faster when elicited by optogenetic stimulation of visual cortex than by real visual stimuli, which follows from the known latencies of transmission from the retina and subcortical visual pathway.

### Application and perspective

Because of the tight homology between the human brain and the NHP brain, the functional characterization of neurons and neural circuits underlying high-level cognition—and cognitive decline—as well as neurological and psychiatric disorders remains heavily dependent on NHP research. Primates, moreover, are the only foveate mammals; thus, they are the only animal model with human-equivalent visual capabilities and oculomotor behaviors [17,18], which makes NHPs a critical animal model for human visual perception, as well as the development and testing of clinical therapies and neural prosthetics. By integrating optogenetics and calcium imaging, AOI offers the ability to precisely determine and manipulate fine functional maps in real time during NHP behavior. One of AOI’s main functions is the precise manipulation of single neurons and simultaneous monitoring of connected neuronal activity to determine the strength of connectivity within neural circuits without unwanted activation of nearby targets.

## Materials and methods

### Ethics statement

All procedures involving animals were in accordance with the Guide of Institutional Animal Care and Use Committee (IACUC) of Peking University Animals, and approved by the Peking University Animal Care and Use Committee (LSC-TangSM-5).

### Experimental animals

Rhesus monkeys (*Macaca mulatta*) were purchased from Beijing Prima Biotech, Inc. and housed at Peking University Laboratory Animal Center. The study used three healthy adult male monkeys 4–6 years of age and weighing 5–7 kg.

### Surgery procedures and implantation of the optical window

Two sequential sterile surgeries were performed on each animal under general anesthesia. In the first surgery, a 16-mm–diameter craniotomy was created in the skull over V1. We opened the dura and injected 200 nl of a 1:1 mixture of AAV1.Syn.GCaMP6s.WPRE.SV40 (CS0564, titer 2.2e13 [GC/ml], Penn Vector Core) or AAV1.hSyn.GCaMP5G.WPRE.SV40 (V4102MI-R, titer 2.37e13 [GC/ml], Penn Vector Core) and AAV9.CamKIIa.C1V1.TS.eYFP.WPRE.hGH (V4545MI-R, titer 1.6e13 [GC/ml], Penn Vector Core) at a depth of approximately 350 μm.

Injection and surgical protocols for each NHP followed from a previous study [36]. Briefly, a small cover glass (6 mm in diameter) with a single pore (0.3 mm in diameter) was used to target the injection pipette and stabilize the cortical surface during each injection. The quartz pipette (QF100-70-7.5, Sutter Instrument, USA) was pulled with a 15–20 μm tip using a laser-based pipette puller (P-2000, Sutter Instrument, USA) and used for virus injections. After injections, we sutured the dura, replaced the skull cap with titanium screws, and closed the scalp. The animal then returned to its cage for recovery and received Ceftriaxone sodium antibiotic (Youcare Pharmaceutical Group Co. Ltd., China) for one week. A second surgery was performed 45 days later to implant the head posts and imaging window. We used a three-point head-fixation design, with two head posts implanted on the forehead of the skull and one on the back. A T-shaped steel frame was connected to these head posts for head stabilization during subsequent imaging and stimulating sessions.

### Behavioral task

We trained each monkey to sit in a primate chair with its head restrained while performing visual fixation and behavioral choice tasks. Eye position was monitored with an infrared eye-tracking system (ISCAN, Inc.) at 120 Hz. Each trial started with the eye fixated on a white 0.1-degree point within a window of 1 degree. Visual stimuli were generated using a ViSaGe system (Cambridge Research Systems) and displayed on a 17-inch LCD monitor (Acer V173, 80Hz refresh rate) positioned 45 cm from the animal’s eyes. Receptive fields of C1V1- and GCaMP-expressing sites were initially localized with small patches of drifting oriented gratings.

We designed a two-block “GO”/“NO GO” detection task in which NHPs made targeted saccades as a means to report perceptually detected Visual Stim or Opto Stim cues (Fig 4A).

In the first block (Visual Stim), a real visual object was presented on the monitor as a GO cue. This visual object was flashed for 22 ms approximately 3 degrees peripheral to the fixation point. The NHPs were trained to generate a saccade within 500 ms of cue onset to obtain a juice reward. The central fixation point remained unchanged for the duration of the trial.

In the Opto Stim block, a 1P laser pulse (with a wavelength of 532 nm, a 1.0-mm diameter, 0.2–2.4 mW/mm^2^, and a duration of 22, 44, or 66 ms) was projected onto the C1V1-expressing cortical site in each monkey as a GO cue instead of a real visual object. We interleaved trials with either mistargeted laser stimulation of the cortex (to an area without C1V1 expression; 8.3% trials; Mistargeted Stim in Fig 4C and 4D) or without laser stimulation (66.7% trials; No Stim in Fig 4C and 4D) as control trials, allowing us to rule out artifacts related to laser operation.

We used a ratio of 1:1 (No Stim:Visual Stim) in visual stimulation sessions, whereas we used a ratio of 8:3:1 (No Stim:Opto Stim:Mis Stim) in optogenetic stimulation sessions. By using fewer Opto Stim trials, we sought to increase the confidence level of the response data by increasing the NHP decision criteria.

### 2P imaging

After a 10-day recovery period following the second surgery, the animals were trained to fixate their gaze on a fixation point. Imaging was performed using a Prairie Ultima IV 2P microscope (Bruker Nano, Inc., FMBU, formerly Prairie Technologies) and a Ti: Sapphire laser (Mai Tai eHP, Spectra Physics) with a 16× objective (0.8-N.A., Nikon). Whereas 920 nm is a commonly used wavelength for 2P imaging in rodents, we used 1,000 nm for our 2P imaging because we found that it achieved higher quality images (and at deeper depths) in our NHP experiments [36]. Fast resonant scanning (up to 32 frames per second) was used to obtain images of neuronal activity (8 fps by averaging every 4 frames). To discriminate GCaMP5G from C1V1–ts–EYFP, GCaMP5G fluorescence was acquired with a 920-nm excitation laser using a 500- ± 12.5-nm filter, whereas EYFP fluorescence was acquired with a 1,040-nm excitation laser using a 525- ± 35-nm filter.

To achieve 2P imaging with a 1,000-nm excitation source, our power density was approximately 7e-5 mW/um^2^ (<50 mW scanning over an 850-μm × 850-μm area), which was approximately 10,000 times less power than the stimulation power level (approximately 0.3 mW/um^2^, 30 mW, 1,070 nm focused on a diameter of 10 μm). Our imaging laser power could therefore not have caused significant photostimulation of C1V1. Even if it did, it follows that its effects must have been approximately 10,000 times smaller than the effects of our intended photostimulation [34].

### 1P optical stimulation

A 532-nm laser was used for 1P optical stimulation. The laser was directly pointed at the target cortical area through the imaging window. Due to the brightness of the stimulation laser and the high sensitivity of the PMTs, a 500-nm band pass (25-nm width) filter was inserted before PMT of green channel during simultaneous 2P imaging. Nevertheless, simultaneous stimulation light could have potentially leaked through the filtering system to cause recording artifacts. We addressed this potential confound by blocking the 532-nm laser light during the scanning of the central image during 2P recordings, using an electronic circuit that powered down the full-field stimulation pulse whenever the imaging scan was within the central 75% of the FOV (S6 Fig).

### Single-cell 2P activation

A secondary femtosecond laser with 1,070-nm wavelength (maximal power, 2.3 watts; pulse width, 50 fs; Fidelity, Coherent, USA) was used on a secondary galvanometer path in the 2P microscope (Ultima IV, Prairie, Bruker, USA) to perform 2P optogenetic activation targeting single cells, while simultaneously recording calcium activity. Spiral regions (5 rotations, 1.2 expansion rate, 0.01 pixel/μs, and 30 repetitions) were defined to point target photo-activation areas (S7 Fig). The laser power was adjusted to 30 mW at the end of the objective with a polarization beam splitter into 1,070-nm femtosecond laser light pathway.

### Image data processing

Customized Matlab software (The MathWorks, Natick, MA) was used to do data analyzing. To correct the image shifts caused by the movement between the objective and the cortex, we first obtained a template image by averaging 1,000 frames in the middle of an imaging session and then realigned images from each session to the template image using a normalized cross-correlation–based translation algorithm.

### Strategy for randomization and/or stratification

The visual stimuli were randomly interleaved during experiments.

### Inclusion and exclusion criteria of any data

No data were excluded during analysis.

### Quantification and statistical analysis

Customized Matlab software was used to perform statistical analysis. As demonstrated in the figure legends, data were presented as individual data points or as mean ± SEM. Number of repetitions for each experiment was also noted within the figure legends.

### Additional resources

The genetic constructs used in this work are available via Addgene.

## Supporting information

S1 Fig Sparse labeling cells in the surrounding regions away from injection centers exhibited membrane localized expression pattern of C1V1. C1V1, ChR1/VChR1.
(TIF)

S2 Fig Responses of neurons to photostimulation at different intensities, from the 30 neurons recorded in Fig 1F, on Day 158 post-infection. (TIF)

S3 Fig Orientation tuning and spatial organization in macaque V1. (A) Left, orientation tuning curves of neurons (*N* = 148) responding to orientation-grating stimuli (*P* < 0.05, ANOVA across six orientations) on Day 158. The orange curve is the average response from all selected neurons (grey), and preferred orientations were rotated to aligned to zero degrees. The curves were fit with circular Gaussian [51]. Right, raw responses (mean ± s.e.m) of four example cells to orientations. (B) Left, spatial organization of orientation selectivity as a function of pixel level in V1 on Day 158. Each color corresponds to the matching orientation in the legend. Image brightness represents the average response strength. Right, orientation pinwheel structure of this cortical area. (C) Left, same as A, collected on Day 292 with *N* = 111 neurons. Right, raw responses (mean ± s.e.m) of the same four cells as in A on Day 292. (D) Same as B, collected on Day 292.
(TIF)

S4 Fig Long-term stability of AOI in V1 of monkey M3. (A) Left, 2P images of a V1 neuronal population on Day 28 and Day 62 after virus injection. Right, the same neuronal population was activated with visual stimuli on Day 28 and Day 62. (B) Left, spatial organization of orientation selectivity in V1 on Day 28 and Day 62. Each color corresponds to the matching orientation in the legend. Image brightness represents the average response strength. Right, orientation pinwheel structure of this cortical area. (C) Orientation tuning curves of neurons (*N* = 55 on Day 28 and *N* = 25 on Day 62) responding to orientation grating stimuli (*P* < 0.05, ANOVA across six orientations). The orange curve is the average response from all selected neurons (grey), with preferred orientations rotated to align at zero degrees. (D) Raw responses (mean ± s.e.m) of three example cells to orientations on Day 28 and Day 62, respectively. (E) Neuronal orientation tuning correlations on Day 28 and 62. The orientation tuned neurons were picked by ANOVA with *P* < 0.05. (F) Distribution of neuronal responses to optical stimulation on Day 28 and 62.
(TIF)

S5 Fig Example cells in 5 evenly spaced layers, corresponding to plots in Fig 2F. Differential images (stimulated baseline [F-F0]) under wide-field photostimulation.
(TIF)

S6 Fig Synchronization control of 2P imaging and 1P photostimulation. (A) 2P image of a cortical area without 532-nm photostimulation. (B) The same cortical area imaged during continuous wide-field photostimulation, resulting in PMT saturation. (C) We powered down the photostimulation whenever the 2P imaging targeted the central 75% of the FOV, allowing the imaging to be sampled artifact-free. (D) Synchronization waveforms for the 2P and 1P activation times waves corresponding to panels A-C. 1P, single-photon; 2P, two-photon; PMT, photomultiplier.
(TIF)

S7 Fig Spatial precision of 2P stimulation after calibration. (A) 5 × 5 grid locations used for calibration. (B) After calibration, a N-type dot array was precisely burned by series of spiral scanning laser (red, 5 rotations, 1.2 expansion rate, 0.01 pixel/μs, and 10 repetitions). 2P, two-photon.
(TIF)

S1 Video V1 neuronal population responses to oriented grating stimuli. V1, primary visual cortex.
(MOV)

S2 Video V1 neuronal population responses to 1P optical stimuli. 1P, single-photon; V1, primary visual cortex.
(MOV)

## Abbreviations

1P: single-photon
2P: two-photon
AAV: adeno-associated virus
AOI: all-optical interrogation
C1V1: ChR1/VChR1
ChR2: Channelrhodopsin-2
CLE: confocal laser endomicroscopy
GCaMP: genetically encoded calmodulin protein
GECI: Genetically Encoded Calcium Indicator
IACUC: Institutional Animal Care and Use Committee
NHP: nonhuman primate
P2A: porcine teschovirus-1 2A
PMT: photomultiplier
ROI: region of interest
V1: primary visual cortex
