# Supplementary figures and images for: Long-term all-optical interrogation of cortical neurons in awake-behaving nonhuman primates
Source: PLoS Biol. 2018 Aug 8;16(8):e2005839. doi: 10.1371/journal.pbio.2005839 (PMC6101413; doi:10.1371/journal.pbio.2005839)

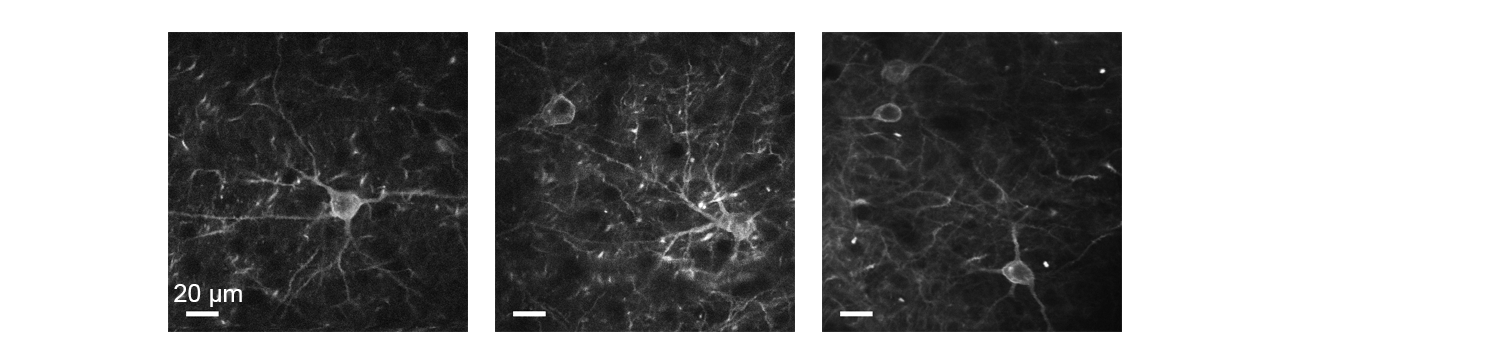

Supplement: S1 Fig — C1V1, ChR1/VChR1. (TIF) [file pbio.2005839.s001.tif]

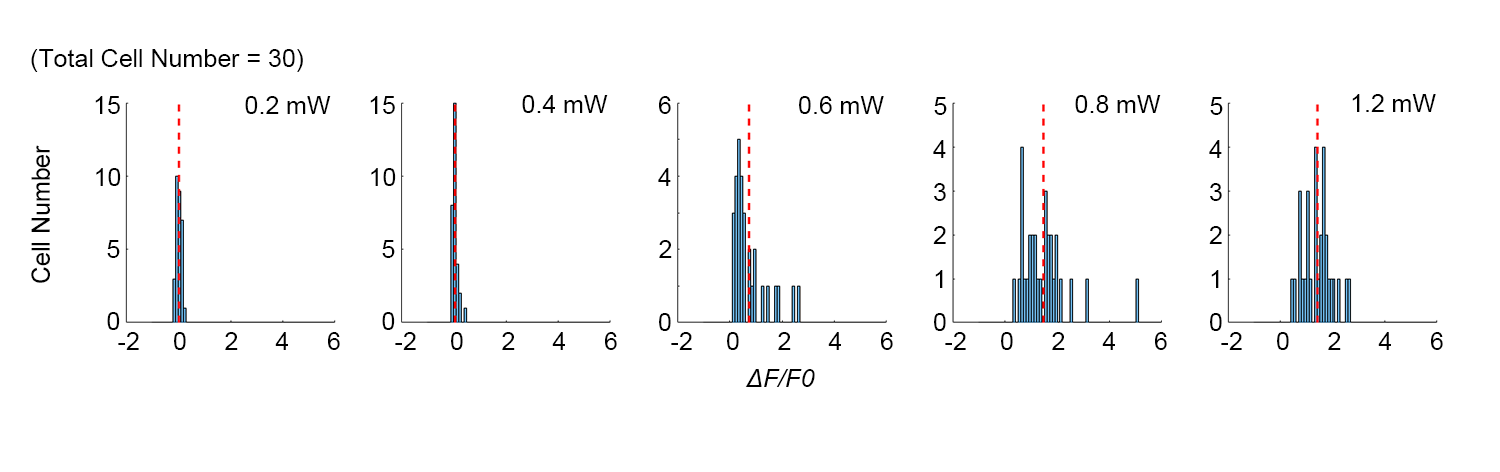

Supplement: S2 Fig — (TIF) [file pbio.2005839.s002.tif]

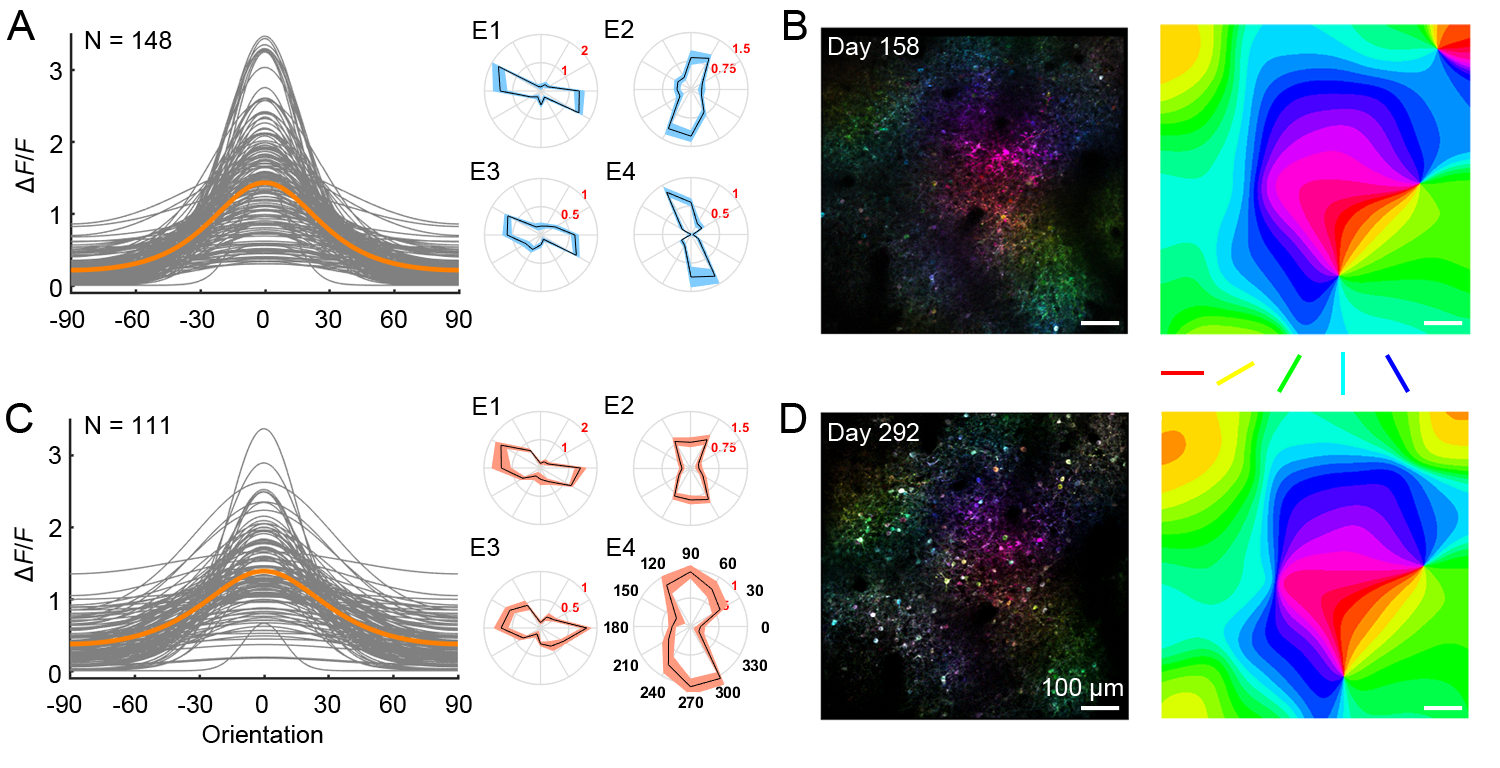

Supplement: S3 Fig — (A) Left, orientation tuning curves of neurons (N = 148) responding to orientation-grating stimuli (P < 0.05, ANOVA across six orientations) on Day 158. The orange curve is the average response from all selected neurons (grey), and preferred orientations were rotated to aligned to zero degrees. The curves were fit with circular Gaussian [51]. Right, raw responses (mean ± s.e.m) of four example cells to orientations. (B) Left, spatial organization of orientation selectivity as a function of pixel level in V1 on Day 158. Each color corresponds to the matching orientation in the legend. Image brightness represents the average response strength. Right, orientation pinwheel structure of this cortical area. (C) Left, same as A, collected on Day 292 with N = 111 neurons. Right, raw responses (mean ± s.e.m) of the same four cells as in A on Day 292. (D) Same as B, collected on Day 292. (TIF) [file pbio.2005839.s003.tif]

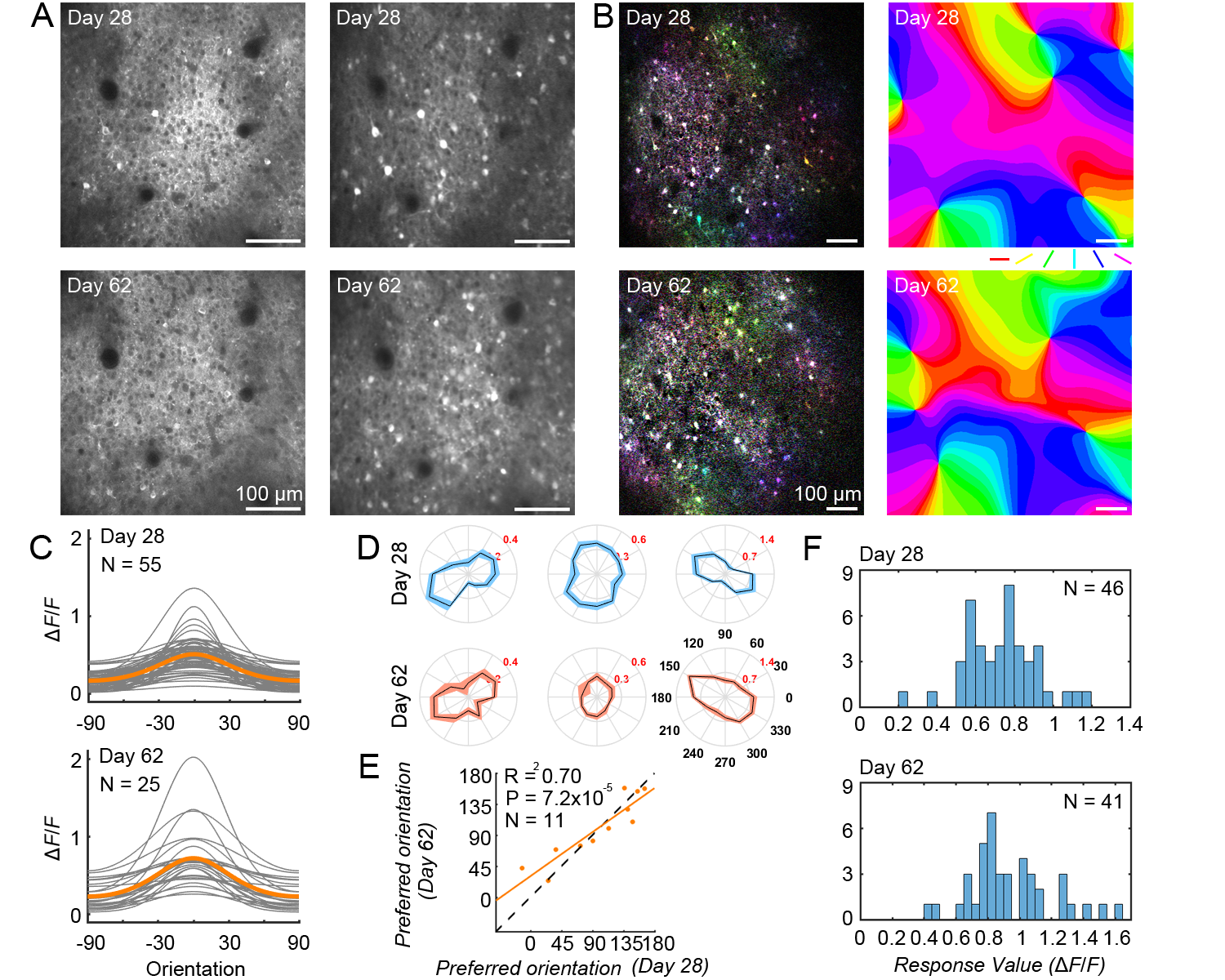

Supplement: S4 Fig — (A) Left, 2P images of a V1 neuronal population on Day 28 and Day 62 after virus injection. Right, the same neuronal population was activated with visual stimuli on Day 28 and Day 62. (B) Left, spatial organization of orientation selectivity in V1 on Day 28 and Day 62. Each color corresponds to the matching orientation in the legend. Image brightness represents the average response strength. Right, orientation pinwheel structure of this cortical area. (C) Orientation tuning curves of neurons (N = 55 on Day 28 and N = 25 on Day 62) responding to orientation grating stimuli (P < 0.05, ANOVA across six orientations). The orange curve is the average response from all selected neurons (grey), with preferred orientations rotated to align at zero degrees. (D) Raw responses (mean ± s.e.m) of three example cells to orientations on Day 28 and Day 62, respectively. (E) Neuronal orientation tuning correlations on Day 28 and 62. The orientation tuned neurons were picked by ANOVA with P < 0.05. (F) Distribution of neuronal responses to optical stimulation on Day 28 and 62. (TIF) [file pbio.2005839.s004.tif]

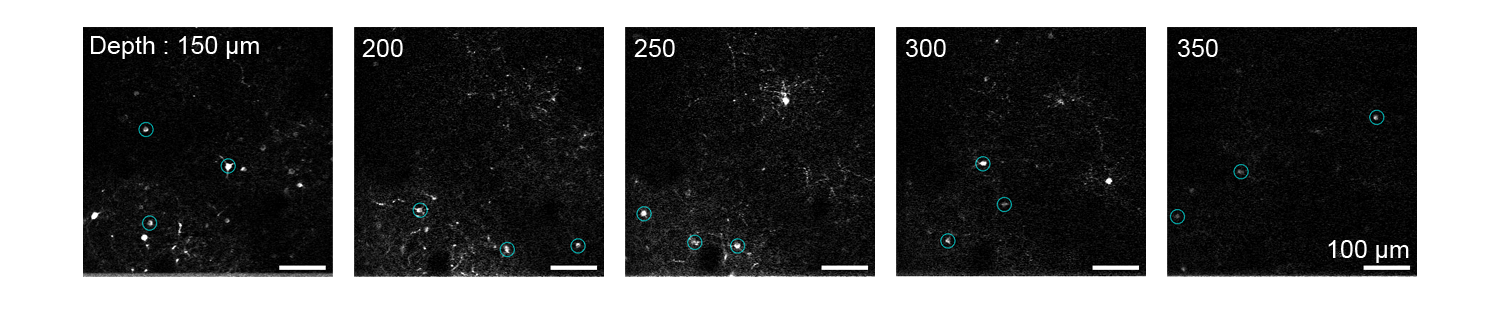

Supplement: S5 Fig — Differential images (stimulated baseline [F-F0]) under wide-field photostimulation. (TIF) [file pbio.2005839.s005.tif]

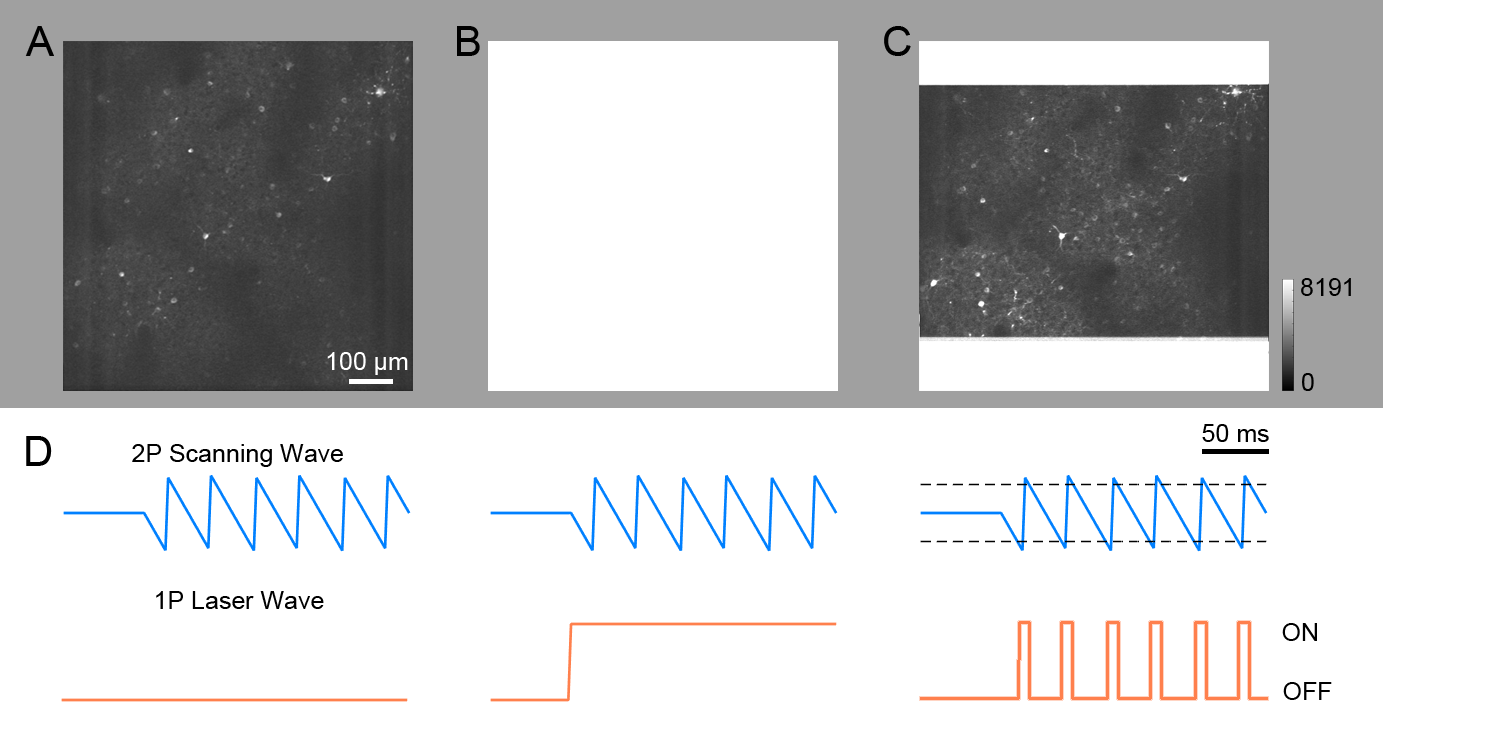

Supplement: S6 Fig — (A) 2P image of a cortical area without 532-nm photostimulation. (B) The same cortical area imaged during continuous wide-field photostimulation, resulting in PMT saturation. (C) We powered down the photostimulation whenever the 2P imaging targeted the central 75% of the FOV, allowing the imaging to be sampled artifact-free. (D) Synchronization waveforms for the 2P and 1P activation times waves corresponding to panels A-C. 1P, single-photon; 2P, two-photon; PMT, photomultiplier. (TIF) [file pbio.2005839.s006.tif]

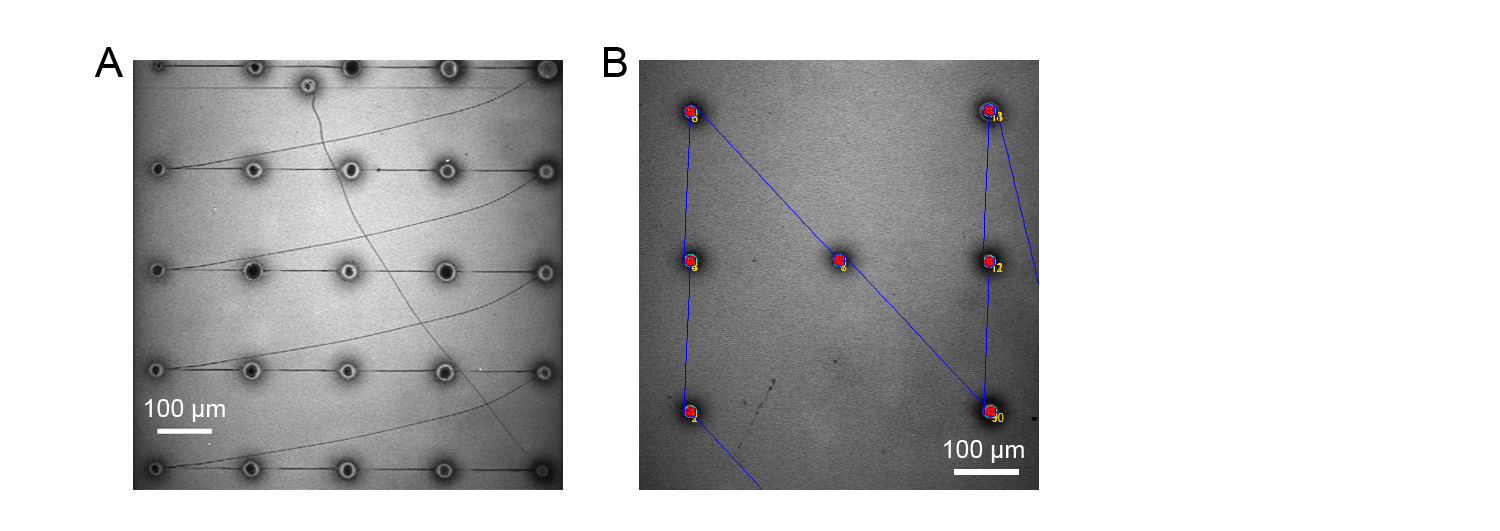

Supplement: S7 Fig — (A) 5 × 5 grid locations used for calibration. (B) After calibration, a N-type dot array was precisely burned by series of spiral scanning laser (red, 5 rotations, 1.2 expansion rate, 0.01 pixel/μs, and 10 repetitions). 2P, two-photon. (TIF) [file pbio.2005839.s007.tif]
